# Acute myeloid leukemia causing acute thrombosis of the coronary arteries: a case report

**DOI:** 10.1186/s13256-022-03280-3

**Published:** 2022-04-12

**Authors:** Meganne N. Ferrel, John J. Ryan, Frederick T. Han

**Affiliations:** 1 University of Utah School of Medicine, 30 North 1900 East, Room 4A100, Salt Lake City, UT 84132 USA; 2 Division of Cardiovascular Medicine, Department of Medicine, University of Utah, Salt Lake City, UT 84132 USA; 3 Division of Cardiovascular Medicine, Section of Cardiac Electrophysiology at the University of California San Diego Health System, 9452 Medical Center Drive, La Jolla, CA 92037 USA

**Keywords:** STEMI, Ventricular tachycardia, Leukocytosis, Fever, Thrombocytopenia, Non-atherosclerotic acute coronary syndrome

## Abstract

**Background:**

This case report demonstrates acute myeloid leukemia causing acute thrombosis of coronary arteries with anterolateral ST elevation myocardial infarction and ventricular tachycardia in an otherwise healthy woman. Few case reports have been documented on patients with concomitant conditions of acute myeloid leukemia and acute myocardial infarction, and it is important to note that prognosis for patients with both is worse than that of either condition. While both conditions together are rare, other non-atherosclerotic causes of acute coronary syndromes are likewise important considerations in the context of myocardial ischemia.

**Case presentation:**

A 59-year-old Caucasian woman with no notable past medical history presented to her primary care provider with 2 weeks of severe fatigue, anorexia, and malaise, associated with chills, night sweats, and myalgias. Peripheral blood smear identified 92% blasts consistent with acute myeloid leukemia and computed tomography identified a right segmental pulmonary embolism and thrombotic infarcts in the spleen, bilateral kidneys, right lung, and liver. Laboratory testing also demonstrated disseminated intravascular coagulopathy. She was admitted to the intensive care unit for treatment and subsequently developed ventricular tachycardia, anterolateral ST segment elevation, acute dysarthria, and nonreactive pupils. Pulseless electrical activity developed with unsuccessful resuscitative measures. The patient died secondary to presumed cerebrovascular and coronary thromboses causing stroke and anterolateral infarct complicated by ventricular tachycardia and pulseless electrical activity.

**Conclusion:**

This case is notable as a case of acute myeloid leukemia causing acute thrombosis of coronary arteries with anterolateral ST elevation myocardial infarction, ventricular tachycardia, and pulseless electrical activity. Prognosis of concomitant acute myeloid leukemia and acute myocardial infarction is poor. Management is challenging due to thrombocytopenia, platelet dysfunction, and systemic coagulopathy, and administration of thrombolytic agents can be fatal. This is an extreme presentation of a case of acute myocardial infarction with disseminated intravascular coagulopathy causing acute coronary thrombosis and sudden death with dramatic electrocardiogram and telemetry findings recorded with rapid progression from normal sinus rhythm to acute myocardial infarction to terminal rhythm.

## Introduction

Acute myeloid leukemia (AML) and acute coronary syndrome (ACS) are rarely reported as concomitant conditions, and the prognosis for patients with both is worse than that of either condition alone. Management is challenging due to thrombocytopenia, platelet dysfunction, and systemic coagulopathy, and administration of thrombolytic agents can be fatal [1].

This case is notable due to rapid progression from normal sinus rhythm to acute myeloid infarction (AMI) to terminal rhythm within a period of 20 minutes. It is uncommon to capture acute dramatic terminal changes of this quality. Additionally, one must maintain a wide differential diagnosis at all times, particularly regarding ACS not of atherosclerotic etiology. Additionally, acute cerebral infarction, arrhythmias, and sudden death during treatment of AML are potentially underrecognized complications that could benefit from inpatient cardiac telemetry and, as needed, hemodynamic monitoring.

The goal of this report is to review non-atherosclerotic causes of ACS through the presentation of a unique case capturing natural progression of ST-elevation myocardial infarction.

## Case presentation

A 59-year-old Caucasian woman presented to her primary care provider with 2 weeks of severe fatigue, anorexia, and malaise, associated with chills, night sweats, and myalgias. She tested negative for coronavirus disease 2019 (COVID-19) with polymerase chain reaction (PCR) test. She denied any prior medical conditions or surgeries. She denied any family history of malignancy or heart disease. After being treated unsuccessfully for a urinary tract infection, she presented a second time to her primary care physician.

Presenting vital signs were as follows: temperature 37.3 °C, blood pressure of 121/74 mmHg, heart rate 117 beats per minute, respiratory rate 18 breaths per minute, and blood oxygen saturation (SpO2) 92% on room air. Physical examination revealed pallor, decreased breath sounds in right base, and left calf tenderness without swelling.

**Fig. 1:**
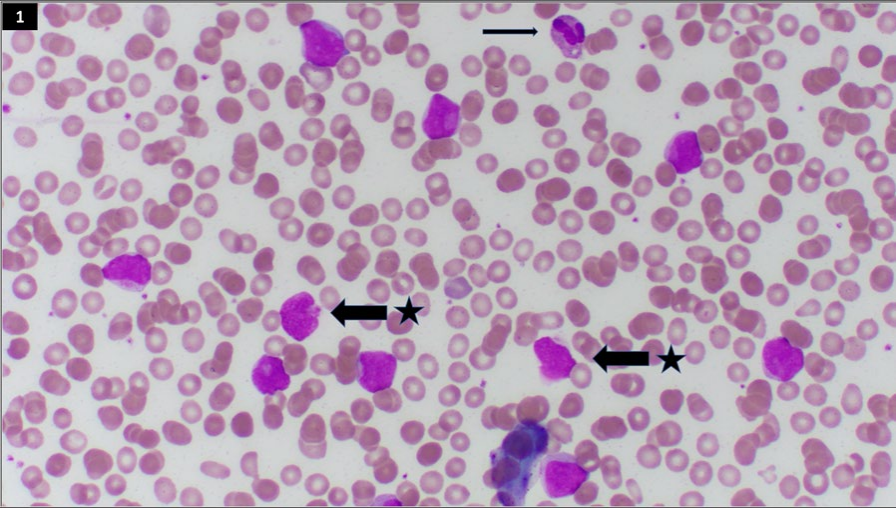
Peripheral blood smear demonstrating blasts (arrows with star) and segmented neutrophil (arrow)

Complete blood count (CBC) showed a white blood cell count of 79,050/µL with hemoglobin of 11.2 g/dL and platelets of < 6000/µL, with a manual differential demonstrating 95% blasts. Computed tomography (CT) of the chest, abdomen, and pelvis identified a right segmental pulmonary embolism and thrombotic infarcts in spleen, bilateral kidneys, right lung, and liver. Peripheral blood smear identified 92% circulating blasts consistent with AML (Fig. 1). Likewise, bone marrow aspirate demonstrated AML by morphology (86.7%) and flow cytometry. Marrow was hypercellular (> 95%) with reduced trilineage hematopoiesis, consistent with AML. Promyelocytic leukemia-retinoic acid receptor α (PML RARA) translocation was negative by fluorescence *in situ* hybridization (FISH). Laboratory analysis also revealed disseminated intravascular coagulopathy (DIC) manifested by prothrombin time 19.7 seconds, partial thromboplastin time 35 seconds, fibrinogen 133 mg/dL, and D-dimer 41.4 µg/mL.

The patient was admitted to the intensive care unit, where she was treated for AML with hydroxyurea 2 g twice daily, all-*trans* retinoic acid until acute promyelocytic leukemia was ruled out, prednisone 1 mg/kg for prevention of differentiation syndrome, allopurinol 300 mg daily, intravenous fluids, and transfusions to maintain hemoglobin > 7 M/µL, platelet > 30,000/µL, and cryoprecipitate to maintain fibrinogen > 150 mg/dL. She was also given fresh frozen plasma to maintain international normalized ratio (INR) < 1.5, and a heparin infusion was initiated once platelet count exceeded 30,000/µL.

**Fig. 2:**
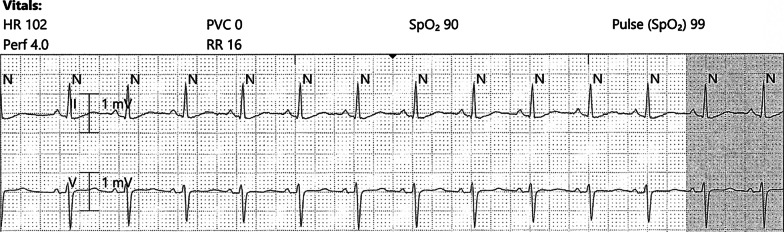
Telemetry. Sinus tachycardia at 07:35

**Fig. 3:**
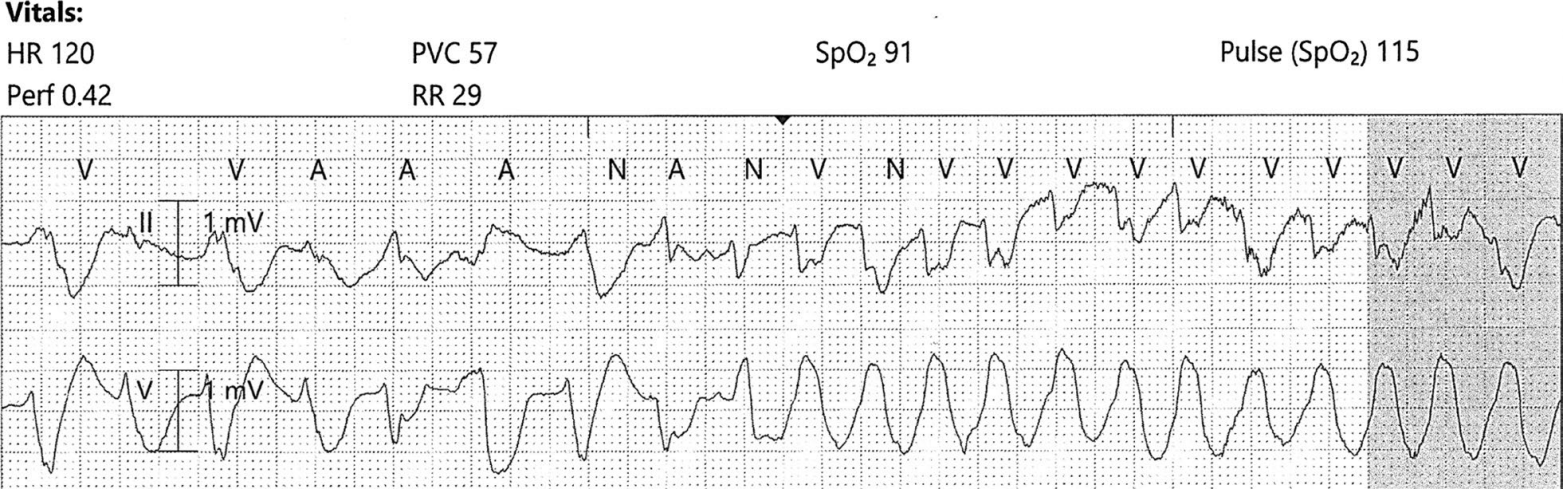
Telemetry. Ventricular tachycardia at 07:37

**Fig. 4:**
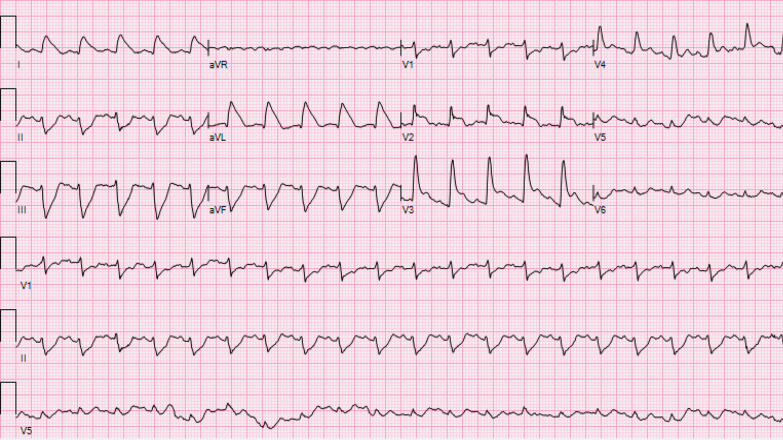
ECG with acute anterolateral ST elevation myocardial injury at 07:48

**Fig. 5:**
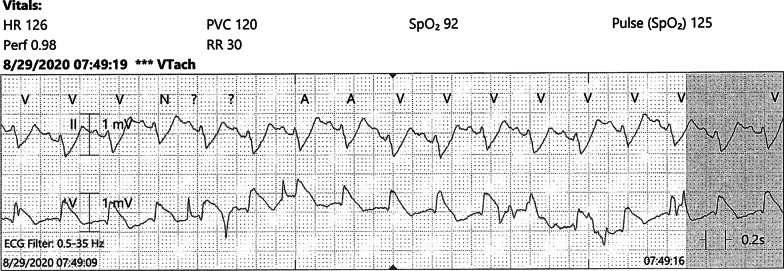
Telemetry at time of acute dysarthria and nonreactive pupils with stroke. Sinus tachycardia at 07:49

**Fig. 6:**
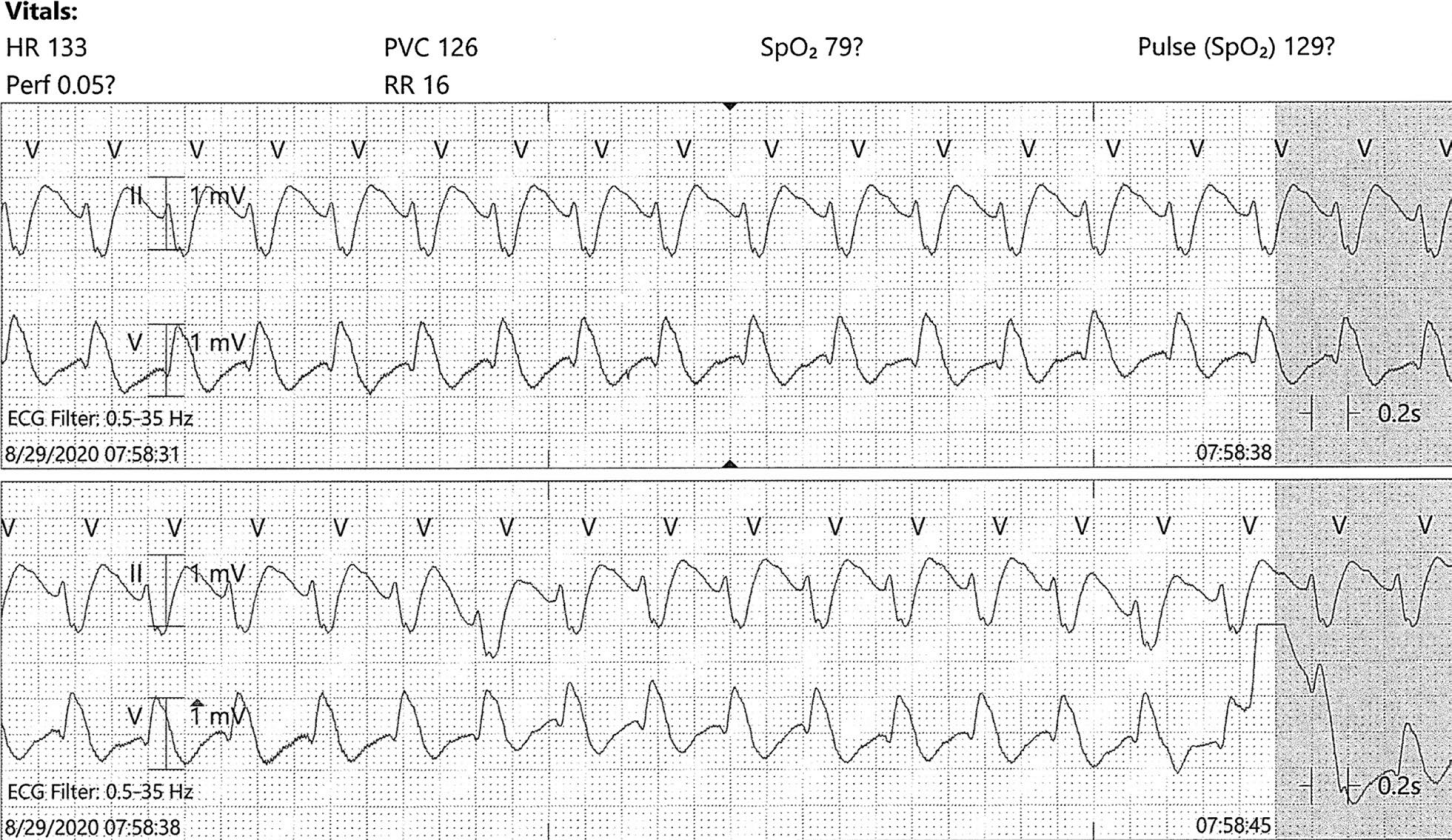
Telemetry with recurrent ventricular tachycardia at onset of pulseless electrical activity arrest

On hospital day 2, the patient developed acute hypoxia with complaints of dyspnea and chest pain. She was tachypneic with respiratory rate of 27 breaths per minute, and telemetry demonstrated sinus tachycardia at 117 beats per minute (Fig. 2). Spontaneous hemodynamically significant ventricular tachycardia (VT) occurred and self-terminated (Fig. 3). After resolution of her VT, an electrocardiogram (ECG) identified the presence of sinus tachycardia with anterolateral ST elevation consistent with acute anterolateral injury (Fig. 4). Soon after, the patient developed acute dysarthria and nonreactive pupils. In the setting of her stroke evaluation, the patient developed recurrent VT with pulseless electrical activity (PEA) (Fig. 5). Resuscitative measures were initiated. However, due to inability to restore spontaneous circulation after 15 minutes of resuscitative efforts, the patient died secondary to presumed cerebrovascular and coronary thromboses causing stroke and anterolateral infarct complicated by VT and PEA (Fig. 6).

## Discussion and conclusions

This case is notable as a case of AML causing acute thrombosis of coronary arteries with anterolateral ST elevation myocardial infarction, VT, and PEA. AML and AMI are rarely reported concomitant conditions, and prognosis for patients with both is worse than that of either condition alone. Management is challenging due to thrombocytopenia, platelet dysfunction, and systemic coagulopathy, and administration of thrombolytic agents can be fatal.

AML causing AMI is a rarely reported condition; however, other non-atherosclerotic causes of ACS are likewise important in the context of myocardial ischemia. The most common causes of non-atherosclerotic ACS include spontaneous coronary artery dissection (SCAD), coronary artery embolism, vasospasm, myocardial bridging, and stress-induced cardiomyopathy [1]. SCAD is most common in younger women and is associated with pregnancy, fibromuscular dysplasia, and coronary tortuosity. Percutaneous coronary intervention in SCAD is associated with higher rate of complications and lower rate of success than in atherosclerotic ACS, with isolated intramural hematoma carrying the highest-risk angiographic phenotype [2]. Coronary artery embolisms likewise should be considered and include etiologies divided into direct, paradoxical, and iatrogenic embolisms. Aspiration thrombectomy should be considered for acute management [3]. Coronary vasospasm can occur at the epicardial or microvascular levels. Diagnosis can be aided by pharmacological provocation testing in the catheterization laboratory. Vasospasm is associated with endothelial dysfunction and smoking and is treated with calcium-channel blockers and nitrates as potential therapies [4]. Myocardial bridging is caused by a congenital anomaly of epicardial vessels. The majority of myocardial bridges are asymptomatic; however, depending on the thickness and length of the bridge, symptoms may arise depending on the thickness and length of bridge, orientation of the bridge relative to myocardial fibers, and presence of loose connective or adipose tissue surrounding the bridged segment. Increased mechanical loads potentially contribute to constrictive vascular remodeling, which can lead to pro-atherosclerotic stimulus distal to the bridge. Due to increased atherosclerotic risk in these patients, antiplatelet therapy is recommended. Medical therapy can utilize β-blockers and calcium-channel blockers for symptomatic patients, and for those refractory to medical therapy, surgical intervention or percutaneous intervention may be considered [5]. Stress (Takotsubo) cardiomyopathy is associated with emotional triggers, but can also occur with physical triggers or even without any evident preceding triggers. Stress cardiomyopathy may be of neurogenic origin since coronary microcirculation is innervated by neurons that originate in the brain stem and mediate vasoconstriction. Early coronary angiography is necessary to distinguish stress cardiomyopathy from ACS since ECG and troponin levels provide insufficient information to differentiate. Medical therapy may consist of angiotensin-converting-enzyme inhibitors or angiotensin-receptor blockers [6]. While these causes of non-atherosclerotic ACS are less common than atherosclerotic etiologies, investigation of these are important in the context of myocardial ischemia.

This case demonstrates the importance of maintaining a wide differential diagnosis at all times, particularly regarding ACS not of atherosclerotic etiology. Potential complications during treatment of AML, including acute cerebral infarction, arrhythmias, and sudden death, are underrecognized, suggesting the need for additional monitoring.

## Data Availability

All data generated or analyzed during this study are included in this published article and its supplementary information files.

## Abbreviations

ACS: Acute coronary syndrome
AML: Acute myeloid leukemia
AMI: Acute myocardial infarction
CBC: Complete blood count
CT: Computed tomography
DIC: Disseminated intravascular coagulopathy
INR: International normalized ratio
PEA: Pulseless electrical activity
SCAD: Spontaneous coronary artery dissection
VT: Ventricular tachycardia
